# Comparison of the abrasive properties of two different systems for interproximal enamel reduction: oscillating versus manual strips

**DOI:** 10.1186/s12903-019-0934-y

**Published:** 2019-11-14

**Authors:** Francesca Gazzani, Roberta Lione, Chiara Pavoni, Gianluca Mampieri, Paola Cozza

**Affiliations:** 1 Department of Clinical Sciences and Translational Medicine, University of Rome ‘Tor Vergata’, Viale Oxford 81, 00133 Rome, Italy; 2 Department of Dentistry UNSBC, Tirana, Albania; 3 Head of the Department of Dentistry UNSBC, Tirana, Albania

**Keywords:** Interproximal enamel reduction, Mechanical strips, Manual strips, SEM analysis

## Abstract

**Background:**

The aim of the present investigation was to evaluate enamel reduction efficiency, abrasive property decay, and enamel effects between oscillating mechanical and manual systems for interproximal enamel reduction (IPR).

**Methods:**

Three oscillating strips and three manual strips were tested on twelve freshly extracted premolars blocked in an acrylic cylinder pot by means of a material testing machine. Each strip underwent one test of 8 cycles (30 s each). Both abrasive tracks and teeth surfaces were qualitative evaluated before and after IPR by means of SEM analysis. Efficiency and abrasive property decay of both IPR systems were investigated by the amount of enamel reduction within the eight-cycle testing. *Independent t-test* was used to evaluate differences in variables between the two systems.

**Results:**

Mechanical IPR system showed higher efficiency in terms of enamel reduction (*p* < 0.005) when compared with manual IPR system (0.16 mm and 0.09 mm, respectively). Quantity of removed enamel decreased throughout the 8 cycles for both systems. Less presence of enamel debris and detachment of abrasive grains were observed on mechanical strips rather than manual strips. SEM analysis revealed more regular surface of teeth undergone mechanical IPR procedures.

**Conclusion:**

Oscillating diamond strips showed more controlled efficiency when compared with the manual IPR system leading to a more regular enamel surface.

## Background

Interproximal reduction (IPR) is a common procedure used in orthodontic treatment [1] in several clinical cases. Main clinical indications include correction of Bolton tooth-size discrepancies, mild or moderate crowding, morphologic dental anomalies, prevention of relapse, and reduction of interdental gingival papilla retraction [1–5]. It is frequently used as part of treatment in combination with clear aligners [6]. Sheridan [1] described Air-rotor stripping (ARS) technique more than 20 years ago as an alternative to extraction borderline cases. Several IPR systems have been developed [6–8] and progressively modified over the years. Recently, many powered IPR systems such as mechanical oscillating abrasive strips or diamond-coated segmented discs have gained in popularity [6–8]. Since these IPR procedures have become more frequent in orthodontic practice, several studies analyzed their effects on enamel surface [9, 10]. Qualitative SEM evaluations [6, 9, 10] showed that IPR systems can affect enamel morphology leaving furrows and scratches. The use of medium and fine manual metallic strips followed by polishing and topical fluoride application were introduced in 1956 by Hudson [11] in order to reduce enamel irregularities produced [12, 13]. Bonetti et al. [14] suggested topical applications of casein phosphopeptide-amorphous calcium phosphate to enhance enamel remineralization after IPR. However, only fewer studies [8, 15–17] analyzed the efficiency of various existing IPR systems. In a recent literature review, Lapenaite et al. [7] compared different IPR systems highlighting their indications, contraindications, and complications. Even if all instruments are effective in reducing interproximal enamel, there are evident differences in terms of efficiency, effects on enamel surface roughness, and technical aspects such as abrasive grain size, application speed, and intensity of use. Moreover, it is important to quantify the amount of enamel that can be removed to prevent residue excessive space, persisting misaligned teeth, or inter-arch discrepancies. High accuracy is required to achieve treatment objectives especially during 3D digitally treatment plans. In regard to this issue, one aim of the present study was to evaluate the efficiency in terms of enamel reduction of two most commonly used IPR systems by means of Instron Universal Testing Machine. A qualitative evaluation of strips and enamel surfaces before and after application of the two different IPR systems was also performed by means of SEM analysis.

## Methods

Three oscillating strips (Intensiv Ortho-Strips L-OS80XC-R/3, Intensiv SA, Montagnola, Switzerland) and three manual strips (Horico Stahlcarbo 304 Medium, Hopf Ringleb & Company, Berlin, Germany) were collected and tested. Twelve teeth were selected from a collection available and obtained over the years from patients who had an extraction therapy at the Department of Orthodontics, University of Rome “Tor Vergata”. Informed consent agreement was signed by all patients for orthodontic treatment and to allow their teeth to be used for research purposes. All extracted teeth were thoroughly cleaned of debris and soft tissue, then conserved and fixed in 4% glutaraldehyde in 0.2-M sodium cacodylate buffer solution at 48 °C. Each tooth root was blocked by acrylic resin (Leocryl, Leone S.p.A. Ortodonzia e Implantologia, Sesto Fiorentino, Florence, Italy) in a cylinder pot, designed and manufactured by a 3D printer (Object Eden260V, Stratasys, Commerce Way Eden Prairie, Minnesota, USA). Each cylinder pot was placed and fixed with screws inside a metallic support designed and manufactured by Leone company (Leone S.p.A. Ortodonzia e Implantologia, Sesto Fiorentino, Florence, Italy).

### Evaluation of enamel reduction efficiency

**Fig. 1:**
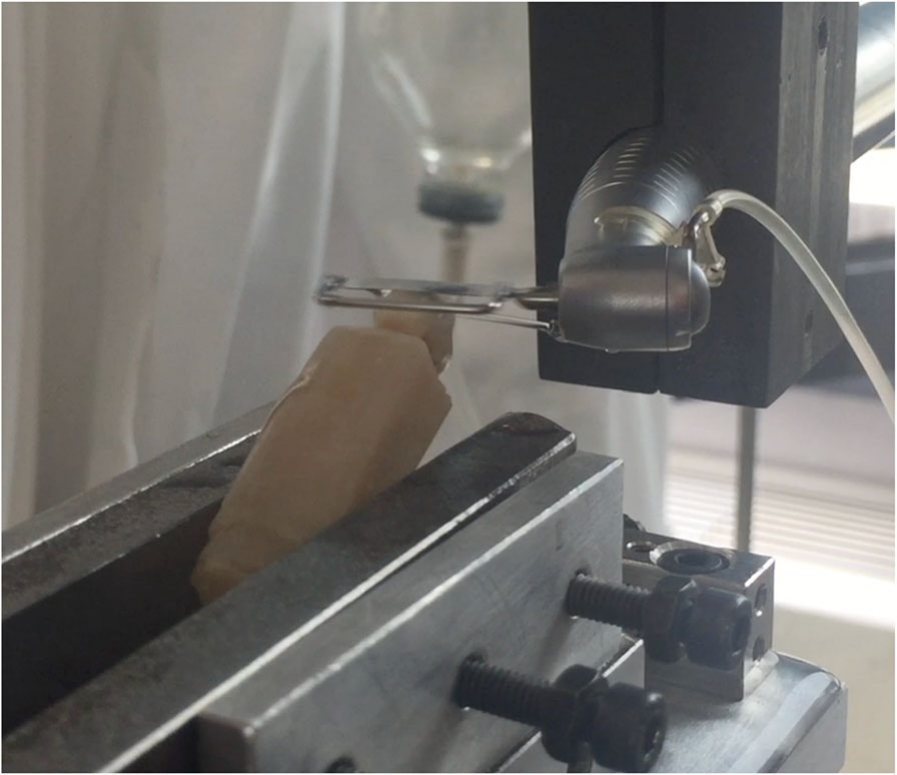
Oscillating diamond strip and contra-angle adapted on the Instron Universal Testing Machine for the experimental analysis

Initially, the amount of enamel reduction achieved in a fixed amount of time by two IPR systems was compared. The experimental analysis was performed by means of an Instron Universal Testing Machine (Model 3365, Instron, Industrial Product Group, Grove City, PA. USA) (Fig. 1). A displacement-controlled method was implemented using Bluehill Software. In the experimental set-up, the reciprocating movement was obtained by means of a contra-angle with 2:1 reduction (Intensiv Swingle, Intensiv SA, Montagnola, Switzerland). Manual strips were also adapted on the oscillating strips framework in order to be applied into designed experimental set up setting the contra-angle at lower oscillations per minute. Before starting the test, a conditioning phase was performed for each strip to correct the lashes of the experimental set-up. The contra-angle with 2:1 reduction was set at the revolutions per minute (RPM) required by the method (40,000 RPM for the mechanical IPR system, resulting in 20,000 oscillations per minute as suggested by the manufacture, and 40 RPM for the manual IPR system, resulting in 20 oscillations per minute simulating manual usage and speed). An adequate water spray (50 ml/s) was activated during the entire test with the mechanical IPR system, as suggested by the manufacturer.

### Description of cycle test setting

Each strip, both oscillating and manual, underwent one test consisting of 8 cycles. Therefore, a total of 24 cycles were performed for 3 oscillating strips and a total of 24 cycles for 3 manual strips. One cycle (30 s) was set according to the following steps:

1. For both systems, contra-angle reciprocating movement started before the data acquisition in order to eliminate any load dissipations (T0, no contact between the strip and tooth surface);
2. With the contra-angle activated, the movable rig of the Instron machine moved down at 0.1 mm/s till the load of 0.1 N (T1, first contact between the strip and the tooth surface);
3. The movable rig moved down of a further 0.8 mm to deflect the strip of 0.8 mm, corresponding to a load of 1 N applied on tooth surface. The strip worked for 30 s (T2, working contact between the strip and tooth surface);
4. At the end of 30 s, the handpiece returned to the starting point (T0);
5. The contra-angle reciprocating movement was stopped and the movable rig of the Instron machine moved down again till the load of 0.1 N (T3, contact between the strip and the tooth surface after stripping).

Each cycle was performed on two untreated tooth surfaces rotating of 90° around the cylinder pot in the metallic clamp support. The down displacement of the movable rig from T0 to T1 position was recorded at the end of each cycle and calculated by Bluehill software. The displacement difference recorded at T3 and T1 was reasonably the dimension of the reduced enamel.

### Evaluation of abrasive property decay of the strips

The effects on strips’ surface structure were analyzed by means of SEM. In particular, both abrasive tracks were analyzed in order to qualitatively evaluate the abrasive grain distribution on the metallic strip matrix, and the presence of enamel debris before and after their use. SEM analysis was performed with a FEI Quanta 200 (Hillsboro, USA) in High Vacuum at 30.00 kV. Images were acquired at 50X, 100X and 200X of magnification. Abrasive property decay for both IPR systems was investigated by evaluating the enamel reduction data described within the 8- cycle testing.

### Evaluation of effects on enamel surface

Also, enamel surface condition was qualitatively evaluated before and after IPR with SEM analysis (Low Vacuum at 10.00 kV) at 30X, 140X, and 300X of magnification. A modified version of a scoring scale previously used by Nucci et al. [18, 19] was adopted to describe enamel surface, and the integrity level of the enamel surface was evaluated as follows:

Score 0: Enamel surface free of scratches and grooves;

Score 1: Scratches and grooves not very accentuated and covering a portion of the surface;

Score 2: Deep furrows with rounded edges evident over the entire surface, without debris;

Score 3: Evident and deep-edged furrows visible on the whole surface and presence of debris on the enamel.

### Statistical analysis

For a standardized effect size of 1 (a clinically relevant change of 0.20 mm with a combined SD of 0.05 derived from a primary pilot test) for the outcome variable enamel reduction in mm, a sample size of 3 strips per group was required for a significance level of 0.05 and test power of 80% [20]. Exploratory statistics revealed that the variable was normally distributed (Kolmogorov- Smirnov test) with equality of variances (Levene’s test). The independent *t*-test was used to evaluate differences in variables between the two systems. Data were analyzed using a statistical software (MS Excel, Micros). Significance level was set at *p* < 0.05.

## Results

**Fig. 2:**
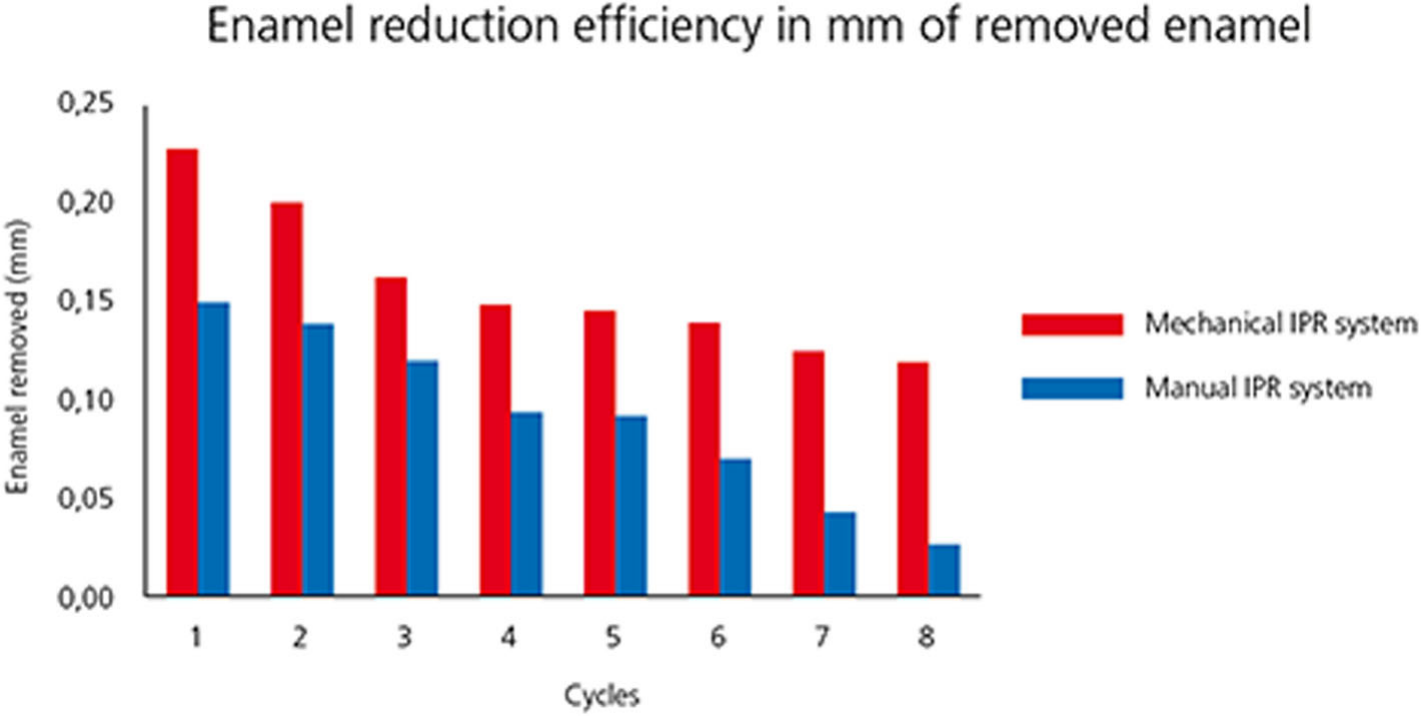
Enamel reduction efficiency (mm) comparing two IPR systems during one cycle test

**Fig. 3:**
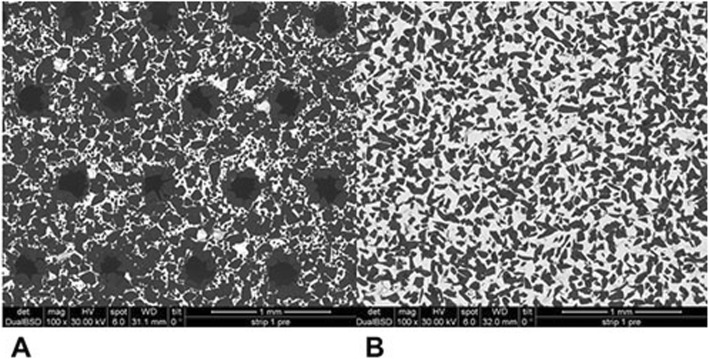
SEM analysis (100X) of non-used mechanical and manual IPR systems

**Fig. 4:**
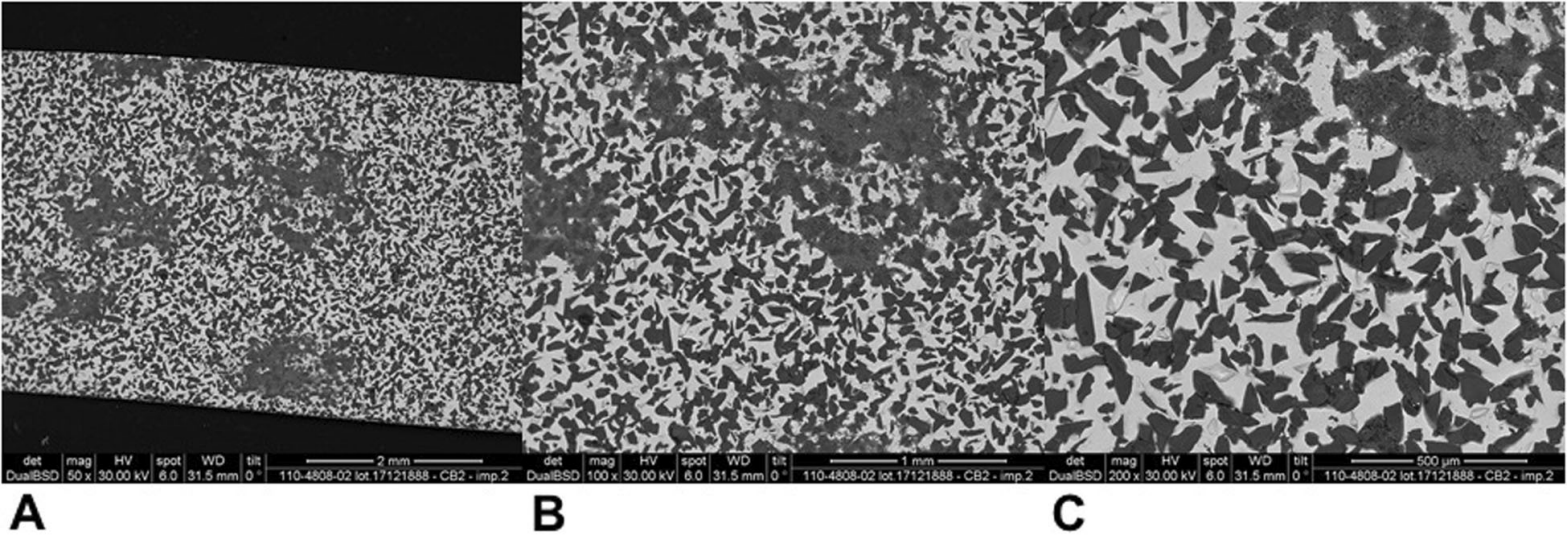
SEM analysis of manual IPR system surface after 8 IPR test cycles. **a** 50X. **b** 100X. **C** 200X

**Fig. 5:**
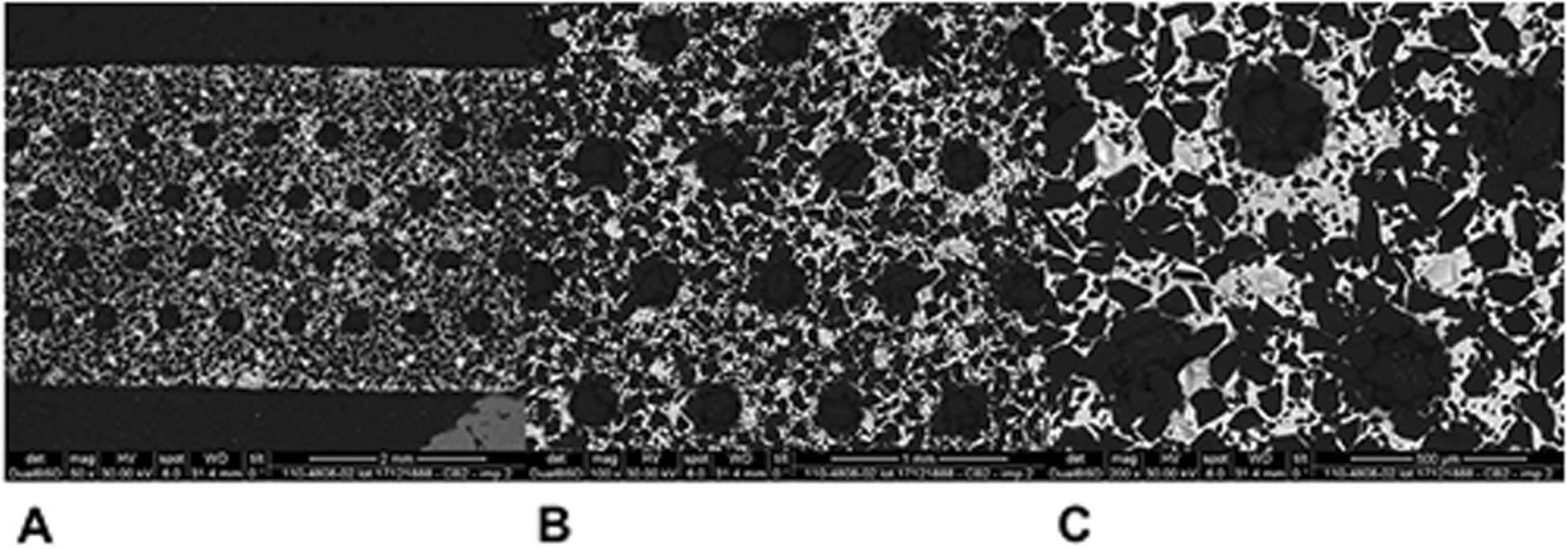
SEM analysis of mechanical IPR system surface after 8 IPR test cycles. **a** 50X. **b** 100X. **C** 200X

**Fig. 6:**
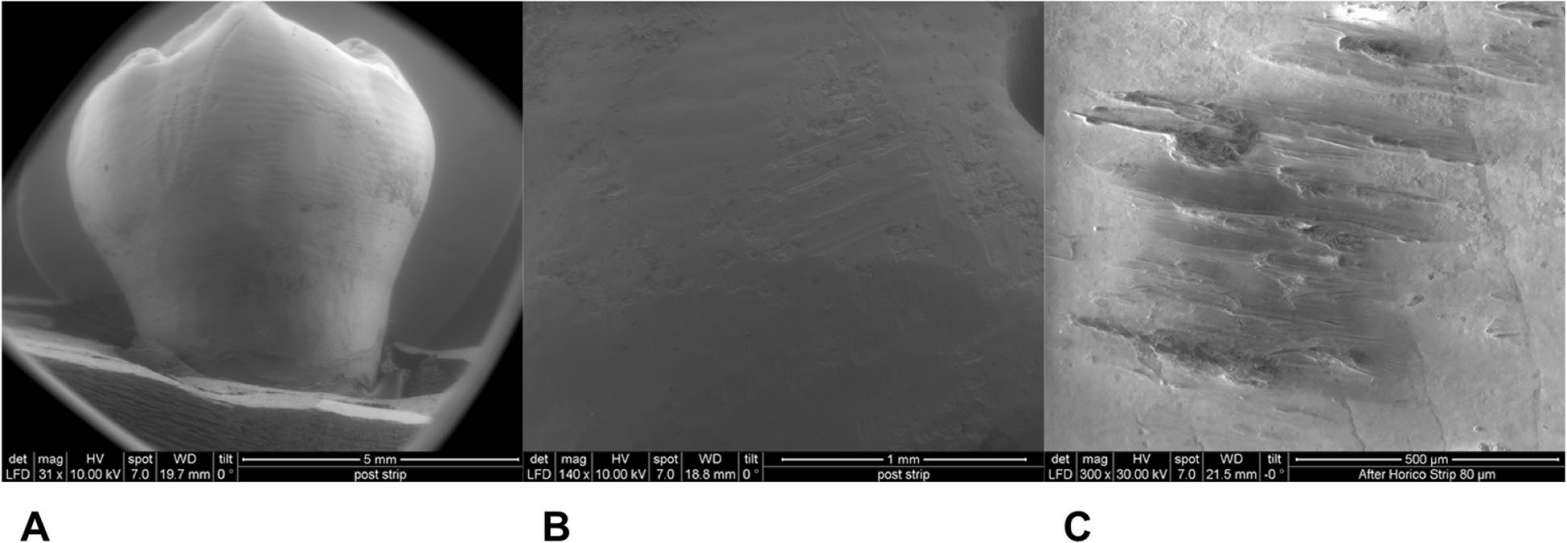
SEM analysis of enamel surface after 8 IPR cycles by means of manual IPR system. **a** 30X. **b** 140X. **C** 300X

**Fig. 7:**
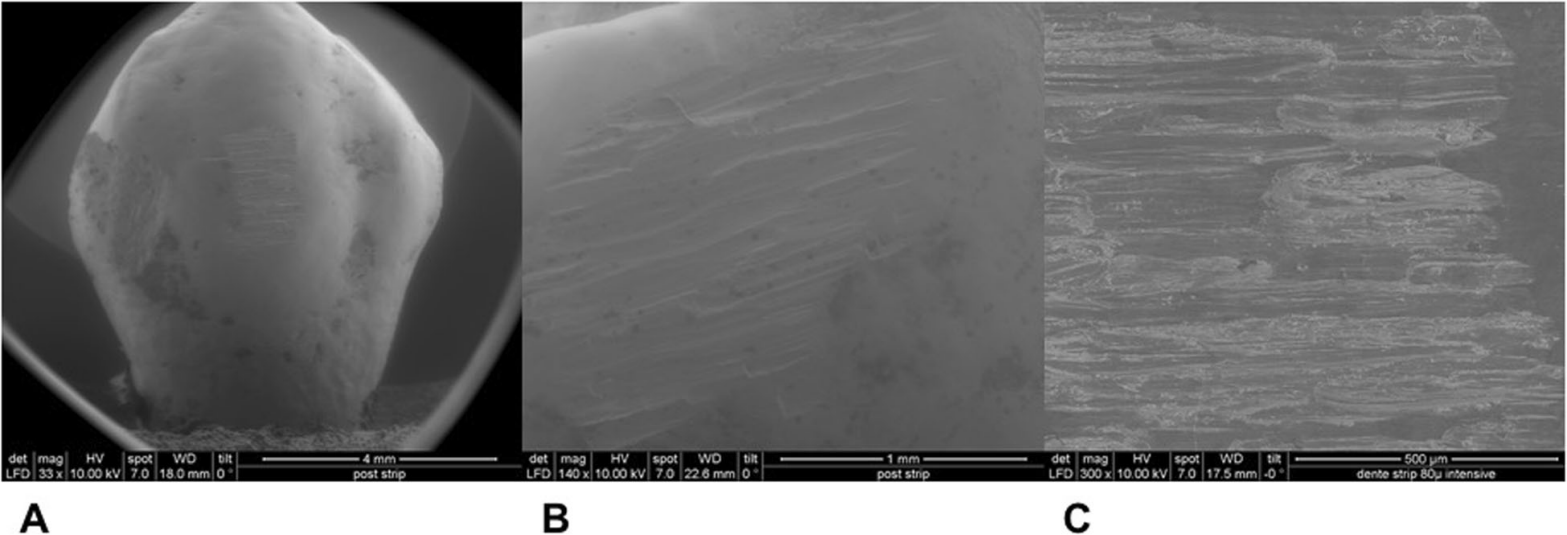
SEM analysis of enamel surface after 8 IPR cycles by means of mechanical IPR system. **a** 30X. **b** 140X. **C** 300X

Mean and standard deviation (SD) of reduced enamel underwent the cyclic test for 8 times are shown in Table 1. Enamel reduction efficiency of two systems throughout the 8 IPR cycles is shown in Fig. 2. Mechanical strips showed higher enamel reduction efficiency in comparison with the manual system. Concerning abrasive property decay, the quantity of removed enamel decreased throughout the 8 cycles for both IPR systems (Fig. 2). Normalization for the two IPR systems was performed according to their respective maximum value of removed enamel: first cycle with mechanical IPR system (0.23 mm of removed enamel) and first cycle with manual IPR system (0.15 mm of removed enamel). The decrease in abrasive properties was significantly less considerable for mechanical IPR system. Before testing, both mechanical and manual strip surfaces showed a metallic substrate with the arrangement of abrasive grains at SEM observation (Fig. 3). Diamond abrasive grains of mechanical strip had a mean dimension of about 80 μm with variable quantity and homogeneous distribution on the metallic substrate. In addition, the abrasive track was characterized by a perforated structure. Manual strip presented aluminum oxide abrasive grains of variable dimension. The surface was continuous without holes. SEM analysis at different magnification (50X, 100X and 200X) showed the presence of enamel debris and the detachment of abrasive grains on both abrasive strips after 8 cycle-tests (Figs. 4 and 5). These two phenomena were less evident on mechanical strips. SEM evaluation (30X, 140 X and 300X) of enamel surface before and after the test is shown in Figs. 6 and 7. All tested surfaces showed scratches and grooves when compared to non-tested surfaces. In particular, SEM analysis revealed different shapes and dimensions of the incisions produced by two different IPR systems. Mechanical IPR system produced more regular surface with a series of light parallel lines with some minor grooves of 1–3 μm and a more uniform enamel coating (Score 1). Manual IPR system revealed a more irregular surface characterized by extended grooves, alternated with enamel ridges and irregular fragment. This configuration corresponds to a Score 2 according to Nucci’s enamel surface classification.

**Table 1:** Descriptive statistics and statistical comparisons (independent-samples t tests) of the enamel reduction efficiency in mm of removed enamel. Mean values obtained from 24 cycles performed for 3 oscillating strips and 24 cycles performed for 3 manual strips

Variable	Oscillating mechanical system (3 strips)		Manual system (3 strips)		Diff.	*P* value	95% CI of the difference	
	Mean	SD	Mean	SD			Lower	Upper
Enamel reduction (mm)	0.16	0.04	0.09	0.04	0.07	0.004*	0.025	0.110

*SD* Standard Deviations, *Diff.* Differences, *CI* Confidence interval

* *p* < 0.005

## Discussion

Increasing demand of alternative procedures to extraction treatments promoted the introduction of several IPR systems [1–8]. Most common ones are manual abrasive strips, mechanical oscillating abrasive strips, diamond-coated segmented discs and rotating diamond burs [1, 6–8, 13]. The effects that IPR can have on the enamel surface have been well documented in literature [9–14]. However, comparative data on the efficiency of different IPR systems are not so common [7, 8, 15–17, 21]. Recently, mechanical oscillating abrasive strips have gained in popularity [7, 22, 23]. Some authors highlighted various advantages of this system in comparison with more traditional ones: avoiding risk of cutting into the soft tissue, possibility of more regular enamel surface, and more predictable results [14, 16, 24, 25]. Several studies [6, 7] concluded that mechanical IPR systems reduce chairside time compared to manual strips. In contrast, manual abrasive strips are particularly indicated for anterior teeth, rotated elements, and recontouring procedures [1, 2]. However, they can result impractical, unproductive, and time-consuming when used for posterior teeth [1–7]. In the present study, both effects on enamel surface and enamel reduction efficiency were investigated. In terms of superficial effects, Arman et al. and Bonetti et al. [9, 14] concluded that all stripping methods significantly roughened the enamel surfaces. According to the recent investigation of Kaaouara Y et al. [26], our results revealed that mechanical oscillating diamond strips produced more regular surface, with light parallel lines and minor grooves than manual abrasive strips. After manual IPR procedures enamel presented a more irregular surface with extended groves, enamel ridges and irregular fragments suggesting some irregularities of manual abrasive track (Figs. 6 and 7). The most considerable presence of enamel imperfections was due to the reduced accuracy and high variability of the abrasive grain size and distribution. Abrasive particle grain size significantly affects the efficiency of dental abrasives, as well as the attainable enamel surface quality [27]. The SEM evaluation revealed some differences in terms of abrasive particle grain sizes between the two strips. Mechanical oscillating diamond strips presented diamonds grain sizes with mean dimension close to 80 μm, while manual abrasive strips were characterized by the presence of grains with variable dimensions. As for enamel reduction efficiency, mechanical oscillating IPR system reduced the inaccuracy of manual IPR systems satisfying precision potentially down to 0.1 mm required by 3D treatment plans such as clear aligners. Enamel removed by mechanical IPR system was of 0.16 mm, whereas the mean value obtained with manual IPR system was of 0.09 mm (Table 1). The higher efficiency of oscillating systems was observed all throughout the 8 cycles (Fig. 2). These findings were correlated with different characteristics and design of abrasive track [7, 15–17]. The perforated structure of the mechanical strips and compulsory water rinsing facilitated the removal of enamel debris enhancing the overall efficiency in combination with higher velocity of the system. On the contrary, the absence of a perforated structure on the manual strips, and the consequent higher enamel deposition on its abrasive track, reduced the abrasive potential and thus its efficiency [23].As for the abrasive property decay, the percentage of decrease in enamel reduction throughout the 8 cycles was significantly lower for mechanical strips, although a constant decrease was observed for both systems. According to a previous study [23], the progressive loss of abrasive properties is due to the presence of enamel debris on the strip surface and the detachment of some abrasive grains. These two phenomena resulted less evident on mechanical strips according to the qualitative characterization of the abrasive surfaces after the test cycles (Figs. 4 and 5). As for enamel structure, both manual and mechanical IPR systems produce furrows and grooves on the enamel surface [9, 10, 27]. Baumgartner et al. [22] concluded that grinding with mechanical oscillating systems resulted in rougher enamel surfaces in comparison to untreated ones. In the present investigation, enamel surface appeared rougher than the untreated control after both IPR procedures. However, mechanical IPR system produced a more regular enamel surface in comparison with the manual IPR system of manual strips. Considering existing literature [6, 14, 16] and the results obtained on the necessity of an adequate polishing after IPR to guarantee a good long-term prognosis, enamel surfaces should be polished after all IPR procedures. A limitation of the present study design was the likelihood of spurious inferences that could affect the results, such as the access to the interproximal point, the severity of crowding, variability in tooth morphology and the bias related to operator ability.

## Conclusions

Mechanical oscillating diamond strips showed more efficiency in enamel reduction and shorter chair-time compared to manual strips. SEM analysis confirmed a more homogeneous abrasive grain-size distribution on the mechanical systems than manual systems. Moreover, the perforated structure and the water rinsing of the oscillating diamond strips facilitated the removal of enamel debris. Mechanical IPR system defined more regular enamel surfaces when compared with the manual IPR system.

## Data Availability

The datasets used and/or analyzed during the current study are available from the corresponding author on reasonable request.

## Abbreviations

3D: Three dimensional
ARS: Air-rotor stripping
IPR: Interproximal reduction
N: Newton
RPM: Revolution per minute
SEM: Scanning electron microscopy

## References

[1] Sheridan JJ (2007). Guidelines for contemporary air-rotor stripping. J Clin Orthod.

[2] Bolton A (1958). Disharmony in tooth size and its relation to the analysis and treatment of malocclusion. Angle Orthod..

[3] Rossouw PE, Tortorella A (2003). Enamel reduction procedures in orthodontic treatment. J Can Dent Assoc.

[4] Zachrisson BU, Nyøygard L, Mobarak K (2007). Dental health assessed more than 10 years after interproximal enamel reduction of mandibular anterior teeth. AJODO..

[5] Zachrisson BU (2004). Interdental papilla reconstruction in adult orthodontics. World J Orthod.

[6] Lombardo L, Guarneri MP, D'Amico P, Molinari C, Meddis V, Carlucci A, Siciliani G (2014). Orthofile(R): a new approach for mechanical interproximal reduction: a scanning electron micro- scopic enamel evaluation. J Orofac Orthop.

[7] Lapenaite E, Lopatiene K (2014). Interproximal enamel reduction as a part of orthodontic treatment. Stomatologija..

[8] Livas C, Jongsma AC, Ren Y (2013). Enamel reduction techniques in orthodontics: a literature review. Open Dent J.

[9] Arman A, Cehreli SB, Ozel E, Arhun N, Cetinsahin A, Soyman M (2006). Qualitative and quantitative evaluation of enamel after various stripping methods. AJODO..

[10] Danesh G, Hellak A, Lippold C, Ziebura T, Schafer E (2007). Enamel surfaces following interproximal reduction with different methods. Angle Orthod.

[11] Hudson AL (1956). A study of the effects of mesiodistal reduction of mandibular anterior teeth. AJODO.

[12] Stecksén-Blicks C, Renfors G, Oscarson ND, Bergstrand F, Twetman S (2007). Caries-preventive effectiveness of a fluoride varnish: a randomized controlled trial in adolescents with fixed orthodontic appliances. Caries Res.

[13] Kirschneck C, Christl J-J, Reicheneder C, Proff P (2016). Efficacy of fluoride varnish for preventing white spot lesions and gingivitis during orthodontic treatment with fixed appliances-a prospective randomized controlled trial. Clin Oral Investig.

[14] Bonetti G, Zanarini M, Incerti Parenti S, Marchionni S, Checchi L (2009). In vitro evaluation of casein phosphopeptide- amorphous calcium phosphate (CPP-ACP) effect on stripped enamel surfaces. A SEM investigation. J Dent.

[15] Grippaudo C, Cancellieri D, Grecolini ME, Deli R (2010). Comparison between different interdental stripping methods and evaluation of abrasive strips: SEM analysis. Prog Orthod.

[16] Zingler S, Sommer A, Sen S, Saure D, Langer J, Guillon O, Lux CJ (2016). Efficiency of powered systems for interproximal enamel reduction (IER) and enamel roughness before and after polishing: an in vitro study. Clin Oral Invest.

[17] Johner AM, Pandis N, Dudic A, Kiliaridis S (2013). Quantitative comparison of 3 enamel-stripping devices in vitro: how precisely can we strip teeth?. AJODO..

[18] Nucci C, Marchionni S, Piana G, Mazzoni A, Prati C (2004). Morphological evaluation of enamel surface after application of two “home” whitening products. Oral Health Prev Dent.

[19] Paganelli C, Zanarini M, Pazzi E, Marchionni S, Visconti L, Bonetti G (2015). Interproximal enamel reduction: an in vivo study. Scanning..

[20] Lerman J (1996). Study design in clinical research: sample size estimation and power analysis. Can J Anaesth.

[21] Zhong M, Jost-Brinkmann PG, Zellman M, Zellmann S, Radlanski RJ (2000). Clinical evaluation of a new technique for interdental enamel reduction. J Orofac Orthop.

[22] Baumgartner S, Iliadi A, Eliades T, Eliades G. An in vitro study on the effect of an oscillating stripping method on enamel roughness. Prog Orthod. 2015; 10; 16:1. 10.1186/s40510-014-0071-8 PMC4384948 25769017

[23] Lione R, Gazzani F, Pavoni C, Guarino S, Tagliaferri V, Cozza P (2017). In vitro and in vivo evaluation of diamond-coated strips. Angle Orthod..

[24] Piacentini C, Sfondrini G (1996). A scanning electron microscopy comparison of enamel polishing methods after air-rotor stripping. AJODO..

[25] Ballard ML (1944). Asymmetry in tooth size: a factor in the etiology, diagnosis and treatment of malocclusion. Angle Orthod.

[26] Kaaouara Y, Mohind HB, Azaroual MF, Zaoui F, Bahije L, Benyahia H (2019). In vivo enamel stripping: a macroscopic and microscopic analytical study. Int Orthod.

[27] Joseph VP, Rossouw PE, Basson NJ (1992). Orthodontic microabrasive reproximation. AJODO..

